# Updating the Role of Reduced Graphene Oxide Ink on Field Emission Devices in Synergy with Charge Transfer Materials

**DOI:** 10.3390/nano9020137

**Published:** 2019-01-22

**Authors:** Minas M. Stylianakis, George Viskadouros, Christos Polyzoidis, George Veisakis, George Kenanakis, Nikolaos Kornilios, Konstantinos Petridis, Emmanuel Kymakis

**Affiliations:** 1 Center of Materials Technology and Photonics & Electrical Engineering Department, Technological Educational Institute (TEI) of Crete, Heraklion 71004 Crete, Greece; polyzoidis@staff.teicrete.gr (C.P.); gveisakis@staff.teicrete.gr (G.V.); kornil@staff.teicrete.gr (N.K.); c.petridischania@gmail.com (K.P.); kymakis@staff.teicrete.gr (E.K.); 2 Department of Mineral Resources Engineering, Technical University of Crete, Chania, 73100 Crete, Greece; 3 Institute of Electronic Structure and Laser, Foundation for Research and Technology-Hellas, N. Plastira 100, Heraklion, 70013 Crete, Greece; gkenanak@iesl.forth.gr; 4 Department of Electronic Engineering Technological Educational Institute (TEI) of Crete, Chania, 73132 Crete, Greece

**Keywords:** field emission, graphene, reduced graphene oxide, polymer composites, graphene ink, cold cathode, Fowler–Nordheim

## Abstract

Hydroiodic acid (HI)-treated reduced graphene oxide (rGO) ink/conductive polymeric composites are considered as promising cold cathodes in terms of high geometrical aspect ratio and low field emission (FE) threshold devices. In this study, four simple, cost-effective, solution-processed approaches for rGO-based field effect emitters were developed, optimized, and compared; rGO layers were coated on (a) n+ doped Si substrate, (b) n^+^-Si/P3HT:rGO, (c) n^+^-Si/PCDTBT:rGO, and (d) n^+^-Si/PCDTBT:PC_71_BM:rGO composites, respectively. The fabricated emitters were optimized by tailoring the concentration ratios of their preparation and field emission characteristics. In a critical composite ratio, FE performance was remarkably improved compared to the pristine Si, as well as n^+^-Si/rGO field emitter. In this context, the impact of various materials, such as polymers, fullerene derivatives, as well as different solvents on rGO function reinforcement and consequently on FE performance upon rGO-based composites preparation was investigated. The field emitter consisted of n^+^-Si/PCDTBT:PC_71_BM(80%):rGO(20%)/rGO displayed a field enhancement factor of ~2850, with remarkable stability over 20 h and low turn-on field in 0.6 V/μm. High-efficiency graphene-based FE devices realization paves the way towards low-cost, large-scale electron sources development. Finally, the contribution of this hierarchical, composite film morphology was evaluated and discussed.

## 1. Introduction

**Figure 1:**
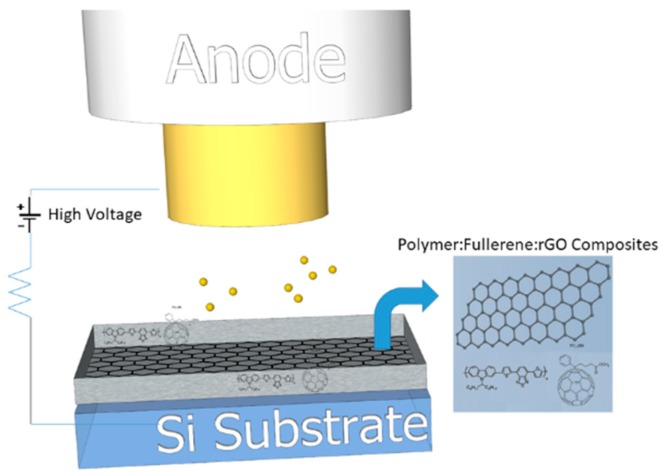
Schematic representation of a field emitter cathode based on hydroiodic acid (HI)-treated reduced graphene oxide (rGO)-charge transfer materials composites.

Upon the existence of a strong electric field that passes through a barrier, electrons are emitted by a sharpened cathode tip. This quantum mechanical tunneling phenomenon is well known as field emission (FE) or “cold cathode” emission. Since higher aspect ratios (height/tip radius) are pursued for the generation of higher FE currents at lower applied electric fields; material properties and cathode configuration are essential for FE characteristics [1]. The wide expansion of FE in various applications including electron guns [2], microwave power amplifiers [3], X-ray tubes [4], neutralizers used in space propulsion devices [5], electron beam lithography [6], and large area field emission sources like flat panel field emission displays (FEDs) [7] stipulates intensive research efforts towards the design and production of electron emitting cold cathodes with improved performance (Figure 1). Aside from that, due to the high-performance requirements of FE materials such as high FE current as well as electrical and mechanical durability in highly distorted geometries, recent FE potential for the realization of integrated flexible devices still constitutes an additional engineering and research challenge. The field enhancement factor β is considered as crucial parameter for the performance of a FE cathode, which can be boosted by increasing the aspect ratio, corresponding to the emitter height over its tip radius. In this context, nanotubes and nanosheets have been considered as emerging materials for FE applications due to their unique 1D and 2D nanostructured geometry, respectively [8,9]. Indeed, the fact that the aspect ratio becomes increasingly high is attributed to their nanoscale tip curvatures and relatively long microscale lengths. Therefore, such nanostructures promote the efficient electron emission at weak applied fields through the generation of a large electric field enhancement.

Although various 1D nanostructures have been placed under the research scope, especially carbon nanotubes (CNTs) have attracted industry’s early interest as emerging FE [8,9,10] for CNT-based FEDs commercialization [11] yet with questionable survival in market. One of the prime concerns is the absence of long lasting FE stability [12] by virtue of the rapid degradation witnessed in CNTs-based emitters, although their electron emission density is tremendous [13]. In addition, in oxygen-rich conditions, CNTs display a perpetual decrease in the FE current density while on the contrary, V_th_ increases [14]. Due to this fact a bottleneck occurs for CNT-based applications, especially in FEDs, under low vacuum or gas purged conditions.

The research rush for graphene and 2D single layer semiconducting materials as inorganic analogues of graphene that demonstrate exceptional physical, optical, and electrical properties has to do mainly with its 2D atomic layer structure [15,16]. However, a low number of studies on the FE properties from such materials, like MoS_2_ sheets, have been undertaken [17]. Moreover, WS_2_ nanotubes also exhibit good FE performance and stability comparable to carbon nanostructure-based field emitters by virtue of their relatively small bandgap, limited number of dangling bonds, mechanical stability, and nontoxicity. Therefore, they pose a promise for a spectrum of technological applications [18]. Yet, WS_2_ nanotubes have to be complemented with a semiconductive polymer, normally P3HT [19], in order to prevent aggregation of the NTs thus enhancing performance of cathodes without polymer. What is more, a polymer matrix is often needed in order to observe protruding nanotubes onto microstructured substrates. This may be ascribed to the low surface tension of NTs/P3HT solution and the large substrate roughness [18]. Alternatively, since the conducting polymer composite serves as an intermediate layer between the WS_2_ nanotube or CNT emitters and a conducting substrate, the electrical contact is improved through the reduction of contact resistance, thereby enhancing overall FE performance [20]. Also, the low electron affinity, wide bandgap excellent transport properties, and flexibility of such polymer-based cathodes have rendered composites of semiconductive polymers with 1D or 2D nanomaterials vital to final related FE optimization [21]. Furthermore, recently, the way to exploit transition metal dichalcogenide (TMD) in FE was cleared with geometrically modulated, CVD-grown MoS_2_, and MoSe_2_ monolayer semiconductors that were suspended with 1D nanoarrays of ZnO was demonstrated, thereby enabling the geometrical modulation and tuning charge transfer [22].

An extended use of graphene nanosheets [15,23] for FE cathodes development is present, thanks to their extraordinary physical, mechanical and chemical properties [18,19,23]. Moreover, the planar 2D structure of graphene renders it to an ideal material for large-area FE devices realization. Since graphene consists of flakes with high aspect ratio as well as sub-nanometer edges, turning graphene into a superior FE is easy, hence allowing the electrons’ extraction at low threshold electric fields with high geometric field enhancement. Although CVD is one of the most successful methods to produce a graphene-based FE, since edge states dominantly contribute to emitting electrons, controlling the orientation of edges perpendicular to substrates is indispensable. In the CVD case, the resulting graphene must go through complex processes such as laser writing [1], plasma treatment [24], or substrate modification [25] so as to create vertical edges, thereby engaging expensive fabrication equipment and long-term procedures.

Other graphene-based FE cathode fabrication routes include liquid phase exfoliation from graphite or exfoliation of graphene from a carbon cloth’s fibers followed by coating graphene solution on a conductive substrate (e.g., Cu) [26,27], electrophoretic deposition (EPD) [27,28], screen printing accompanied with selective photoetching techniques [29], or even blade and ultrasonic spray coating [30] to name just few of them.

**Figure 2:**
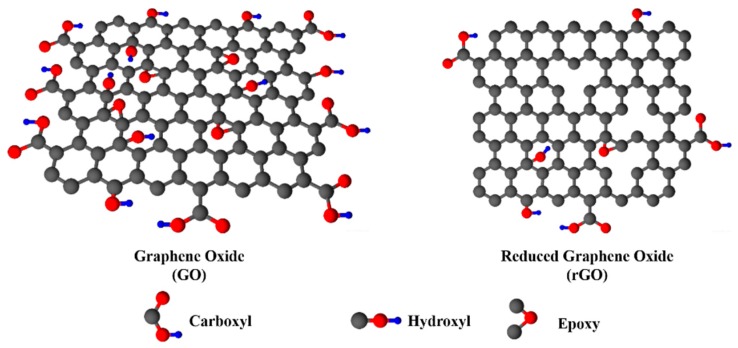
Depictions of GO and rGO structures.

Apart from the previous techniques, chemical oxidation of bulk graphite by Hummers is a strong candidate for resulting to the high yield production of graphene oxide (GO). For instance, a low-cost and successful chemical method to synthesize FE-applicable reduced GO, the annual-ring graphene (ARG) method [31], which demonstrates the advantage of facile synthesis and yields abundant vertical edges on the cross-section of ARG, hence equaling or even surpassing FE performances of CNT [13], cannon-structured graphene film [32] and rGO/MnO_2_ composite [33], not to mention the ultralight weight and flexible nature of the cathode. In general, albeit GO has a similar atomic single layer structure to graphene, the existence of oxygen functional groups attached onto sp^3^ hybridized carbon atoms impacts on its conductivity loss, mainly due to the presence of epoxy and hydroxide groups on its basal plane [34], as illustrated in Figure 2. In order to come up against this side effect and to partially recover the conductivity, various reduction methods have been developed to reduce GO in the form of rGO towards the realization of efficient electronic functionalities.

Chemical reduction can in general be achieved in both liquid and gas phases [35]. Chemical reducing methods include agents that range from hydrazine [36,37,38,39] and Hydrogen Iodide gas [40] until even ascorbic acid [41], KOH, or NaOH [42]. Aside from chemical, thermal reduction at high temperature under inert atmosphere [34,43], and application of either electric [44], or electromagnetic field [45] are also successful reduction routes.

Before moving into plain comparison of FE parameters, considering the way of GO reduction is essential to deeper understanding the correlation between FE and GO, while taking into account only the geometric aspect ratio is insufficient on its own. The oxygen/carbon atomic ratio of the rGO lattice, determined by X-ray photoelectron spectroscopy (XPS), indicates that GO lattice parameters vary depending on the applied reduction technique. In the meanwhile, the Work Function (WF) among different rGO samples, validated by UPS measurements, gave similar trends and thus resulted in different FE characteristics. In general, lower content of oxygen-containing groups exhibits an enhanced FE performance mainly thanks to the WF increase [46]. Besides, the electron transfer at the interfaces between the substrate and the cathode material is expected to be highly promoted, since the states’ density in the cases of oxygen elimination (e.g., HI-assisted rGOs) is also anticipated to be higher.

The role of GO concentration, namely flake density, impacts on the degree of reduction and morphology. Proper partial reduction leaves acidic groups on rGO surface and the polar nature of GO remains and accounts for stable, homogeneous dispersions in solvents, more notably in DMF and NMP [47]. In the case of low rGO-flake density films, the number of emitters is diminished, and the geometrical characteristics of the emitters are resultingly of minor importance are diminished, thus leading to a sharp decline in their performance as cold cathodes. On the one hand, the enhanced roughness at low rGO concentration suggests random sheets orientation onto the planar substrate, while on the other hand, screening effects are observed in the case of high-density films [48,49] followed by an understated emission performance.

On the other hand, polymer concentration affects morphology in a significant manner, while a polymer:rGO concentration ratio exists for optimized FE performance. For high polymer contents fewer rGO sheets are exposed to vacuum, while the thermal conductivity may be four times higher than ambient air for the case of P3HT:rGO, hence easing heat dissipation and achieving higher stability contrary to bare rGO [21]. For low polymer content, rGO flakes may become more preferentially oriented parallel to the substrate [36] and fewer emitting edges may be exposed to vacuum; as a result, the emission performance would be decreased again.

**Figure 3:**
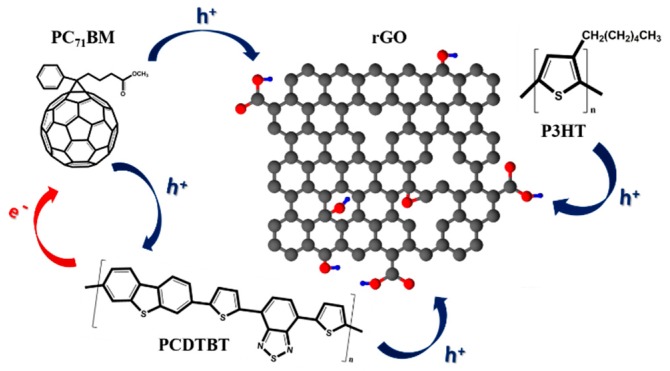
Comprehensive depiction of current conduction routes encountered in the studied composite cases.

Edge density was also increased upon the deposition of an additional rGO layer onto the composite films, thanks to the smoother surface of the top rGO layer in the case of higher rGO content. In other cases, where the polymer concentration was higher, rGO arrays protruded from the polymer bulk, favoring the synergy of two conduction systems: conduction between rGO flakes of the same array, as well as between rGO flakes and the polymer [50]. The relevant charge transfer mechanisms are illustrated in Figure 3. Upon the additional rGO layer deposition onto the polymer:rGO composite layer, the DMF solvent of the rGO dispersion (or any other suitable solvent that might be applied) may dilute some polymer of the underlying layer, thereby a part of the rGO flakes and/or arrays penetrate the polymer. Therefore, since the polymer:rGO ratio increases together with the polymer content, rGO flake density into the polymeric scaffold gets consequently minimized, thus leading to a relevant deterioration in the edge density of rGO exposed to vacuum.

Laser processing plays a crucial role in fabricating 2D-based field emitters. To begin with, structuring Si substrate to create conical, well-separated spikes that are perpendicular to substrate is crucial to good GO deposition and edge protrusion. The microspike arrays are reportedly fabricated by femtosecond (fs) laser texturing of n-type Si, usually followed by consequent removal of grown oxides by HF on spikes’ surface [36]. Secondly, direct laser writing (DLW) is an additional promising technique for the rapid and facile fabrication of graphene for various applications [1,51]. Proper laser treatment gives rise to preferential protrusion of rGO sheets from the substrate and in series to FE characteristics superior to those of pristine rGO. rGO bundles align themselves perpendicular to the substrate, while at the same time sharp graphene edges are protruding out of the bundles. Epidermal treatment of rGO prevents thermal damaging of substrate. As a side advantage, DLW retains the performance of the developed flexible cathodes upon extensive bending conditions, thereby having good potential for graphene-flexible FE cathodes. Finally, selective laser reduction of GO has received attention due to its high potential [52,53] in a diversified field of applications, including FE among them.

This study aims to offer a deeper insight on how three optimized FE enhancement routes contribute to rGO-based FE enhancement. Section 2 discusses the realization of HI-assisted reduction of GO and subsequent rGO ink formation, as well as the optimization process of P3HT:rGO, PCDTBT:rGO and rGO-PCDTBT:PC_71_BM composites by diversifying rGO relative concentrations, whereas final FE cathode fabrication steps are presented. Results are presented in Section 3, and compared between each other in Section 4, while subjecting to discussion concerning FE enhancement in terms of morphology, solvent, reduction agent, or material type (plain rGO ink, polymer:rGO, and polymer:fullerene blend:rGO composite ink). Introducing a PCDTBT:PC_71_BM blend to FE cathodes has to the best of our knowledge never been suggested before. For the first time a HI-assisted rGO has been introduced into FE cathodes in synergy with charge transfer materials’ blends; fullerene-based materials with high electron affinity and polymers are studied. The obtained results are superior to those of previous studies with rGO and polymers. [21] Moreover, the different impacts of fullerene and polymer constituents according to their relative concentrations, physical and chemical contribution are interesting subjects for further investigation.

## 2. Materials and Methods

### 2.1. Preparation of Starting GO Powder

GO was prepared from purified natural graphite powder (Alfa Aesar, ~200 mesh) according to Hummers’ method [54,55]. H_2_SO_4_ (40 mL) was added to 500 mg graphite and stirred for several minutes, followed by the addition of NaNO_3_ (375 mg). Further stirring took place inside a cooled beaker for 2 h, during which 3 g of KMnO_4_ was added. Further stirring step lasted for other 4 h when the reaction mixture was left to reach room temperature before being heated at 35 °C for 30 min. It was then poured into a flask containing deionized water (50 mL) and the stirring temperature was further raised to 70 °C for 15 min. The mixture was then decanted into 250 mL of deionized water; the unreacted KMnO_4_ was removed by subsequent addition of 3% H_2_O_2_ (2 mL). The obtained graphite oxide was purified by repeated centrifugation, which initially yielded a sediment of acidic nature; thus, deionized water and HCl were added to capture sulfate ions so that sediment reaches pH 7. The final product was oven-dried and sieved.

### 2.2. Production of rGO Ink

**Figure 4:**
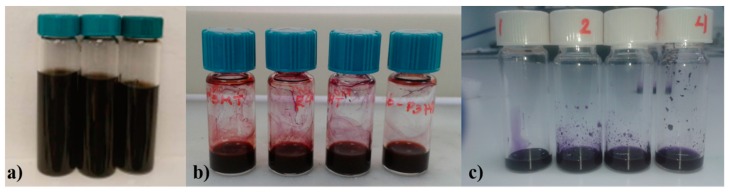
(**a**) Unmixed rGO ink, (**b**) P3HT:rGO, and (**c**) PCDTBT:rGO blends in controlled volume ratios.

A highly efficient HI/AcOH assisted reduction route was chosen to be implemented, as it is described in our previous publication [56]. In a similar manner, our GO was reduced using a mixture of hydriodic acid (HI, 55%)/acetic acid (AcOH). More specifically, the as-prepared GO powder (0.1 g) was sonicated in AcOH (37 mL) for 2 h. Then HI (2 mL) was added and the mixture was stirred at 40 °C for 40 h. Afterwards, the product was isolated by filtration and purified through a three-step washing procedure: (1) aqueous solution of saturated sodium bicarbonate (NaHCO_3_, 3 × 2.5 mL); (2) DI water (3 × 2.5 mL); and (3) acetone (2 × 2.5 mL). Finally, the resulting rGO was dried at 60 °C in a vacuum oven overnight. In the aftermath, the reduced GO powder underwent pulverization procedure with mortar and pestle. Next, rGO powder (20 mg) was added in three vials containing THF (20 mL), DCB (4 mL), and DCB:CB (3:1 *v*/*v*, 20 mL), respectively, in order to predetermine the initial concentration to 1 mg/mL. Then, they were sonicated using an ultrasonic probe (Hielscher UP200Ht) for 1 h and centrifuged at 4000 rpm for 45 min (Allegra X-22) to remove the large aggregates. Afterwards, the supernatants were carefully collected with a pipette and surfactant-free viscous rGO inks were formed (~0.5 mg/mL in THF, ~0.8 mg/mL in DCB, and ~0.85 mg/mL in DCB:CB). The resulting rGO inks are depicted in Figure 4a.

### 2.3. Preparation of rGO-Polymers Composite Inks

P3HT (10 mg) and PCDTBT (4 mg) were dissolved in THF (1 mL) and DCB (1 mL), respectively. Polymers:rGO composite inks were prepared by adding rGO ink to the P3HT and PCDTBT solutions, respectively into different combinations of relative volume ratios (0:100%, 20:80%, 40:60%, 60:40%, and 80:20%). In order to prepare homogeneous polymers:rGO composite inks, the mixtures were further sonicated in an ultrasonic bath (Elmasonic S30H) for another 1.5 h and finally were left undisturbed for 15 min, in order to settle down any agglomerates. Polymers:rGO composite inks are depicted in Figure 4b,c, respectively.

### 2.4. Preparation of rGO-PCDTBT:PC_71_BM Composite Ink

On the other hand, the polymer–fullerene powder was unable to dissolve adequately 4 mg/mL PCDTBT:PC_71_BM solution in THF; therefore, a mixture of DCB:CB (3:1 in volume ratio) was finally applied to dissolve successfully the solid components with sufficient magnetic stirring. PCDTBT:PC_71_BM were dissolved in DCB:CB (3:1 *v*/*v*, 1 mL) with 1:4 (4 mg:16 mg) ratio and stirred overnight at 70 °C. At next, PCDTBT:PC_71_BM:rGO composites were realized by adding rGO ink, prepared in DCB:CB, to the PCDTBT:PC_71_BM blend in different combinations with relative volume ratios (0:100%, 20:80%, 40:60%, 60:40%, and 80:20%). In order to prepare homogeneous PCDTBT:PC_71_BM:rGO composite inks, the above described procedure was also followed.

### 2.5. FE Cathodes Realization

Silicon wafers (n^+^ Si) were subsequently rinsed with acetone and isopropyl alcohol. Composite inks were then realized and coated on top of substrate surface with the drop casting method. Subsequent rGO layer was later on coated in the same manner. All films were placed in an oven for an adequate time until the full removal of the solvents. Final FE cathode structure is illustrated in Figure 1.

### 2.6. Fowler–Nordheim Theory

The FE properties of graphene can be determined using the Fowler–Nordheim law [26]. In brief, the relationship of FE current density (*J*) with the applied electric field E is demonstrated below as Equation (1):

(1) $$J=\eta \;\alpha \;\frac{{\left(\beta E\right)}^{2}}{\varphi }e^{-b\frac{\varphi 3/2}{\beta E}}$$

where constants alpha (α) and b correspond to $\alpha =1.54\cdot 10^{-6}A\;\mathrm{eV}\;V^{-2}$ and $b=6.83\cdot 10^{3}\;eV^{-3/2}V^{-1}$, *φ* represents the work function of the material, g is related with the emitters’ geometrical efficiency, beta (β) is the field enhancement factor, and E corresponds to the macroscopic electrical field in V/μm. E is calculated from E = V/d, in which V denotes the applied potential and d the interelectrode distance. According to Fowler–Nordheim equation, the part of the ln(J/E2) − (1/E) plot that is linear gives proof of electron movement through the tunneling barrier of the emitter in the specific area.

The slope of the previous equation (kFN) derives the field enhancement factor β according to Equation (2):

(2) β=b φ3/2kFN

Additional parameters for assessing FE quality include the E_to_ (turn-on electric field), E_th_ (threshold of electric field), the intensity of luminescence, and the stability of emission. What is sought is minimum values of E_to_ and E_th_, maximum values of the field enhancement factor and emission current density, as well as maximum stability so as to yield an ideal field emitter.

## 3. Results

### 3.1. Field Emission Measurements

FE measurements were executed under vacuum of less than 10^−6^ Torr. Samples were used as cold cathodes in a short circuit-protected planar diode system, as depicted in Figure 1. More details regarding the experimental FE setup can be found elsewhere [48]. Current density−voltage (*J−V*) curves were obtained while the interelectrode distance, d = 200 μm in our case, was controlled by a stepper motor. It is pointed out that FE characteristics remained unaffected by the anode location. In order to confirm the stability of the devices, as well as *J–V* reproducibility, continuous emission cycles were performed. A high voltage (HV) source (PS350-SRS) supplied a voltage with variable sweep step between electrodes. A digital picoammeter (Keithley 485) was used for the sake of measuring FE current. The emission current stability versus time was investigated by monitoring the emitted current density rate over a long time period of consecutive operation. 

#### 3.1.1. First Case: HI-Reduced GO Emitter

**Figure 5:**
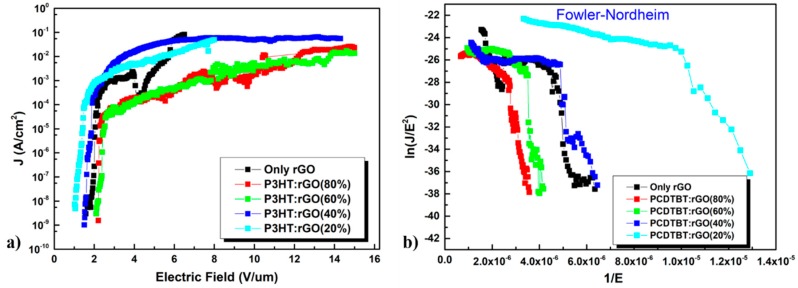
(**a**) Logarithmic plot of the current density, measured as a function of the electric field E (*J*–*E*), obtained by different concentration ratios of rGO ink in composite n^+^-Si/P3HT:rGO field emitters. Lowest electrical field threshold appears for the P3HT:rGO(20%) case. (**b**) Fowler–Nordheim curves of the *J–E* plots of FE with different concentration ratios of rGO ink in composites n^+^-Si/PCDTBT:rGO.

**Figure 6:**
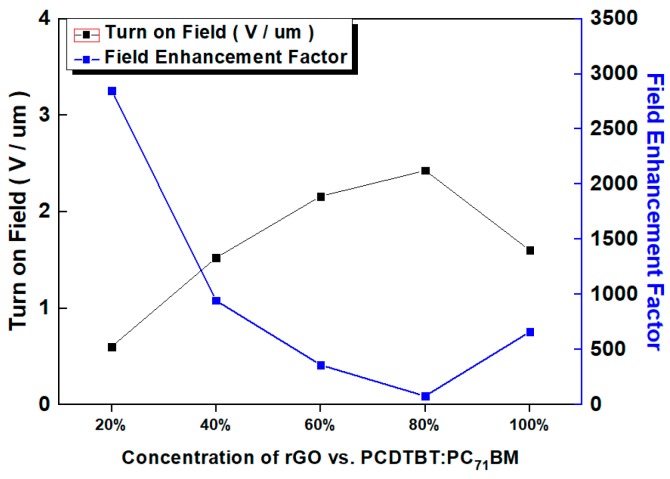
Variation of the turn on field (black line) and the enhancement factor (blue line) in different concentrations of PCDTBT:PC_71_BM:rGO and P3HT:rGO composite inks.

A set of FE cathodes with different rGO ink ratios was realized and subsequently characterized. Figure 5a,b and Figure 6 display the FE response of the best FE cathodes per each rGO ink ratio. Indeed, the beta factor stated at a value of 660, while the E_th_ value was 1.6 V/μm. Numerical results are shown in Table 1.

#### 3.1.2. Second Case: Polymer:rGO Composite Emitter

Among all different volume ratio variants, that of 80% polymer (P3HT or PCDTBT):20% rGO gave the best results. Here, a set of five different ratio batches have been fabricated and tested, while each case includes five fabricated cathode samples. Accordingly, Figure 5a depicts a comparative chart of the best samples per each one of all five ratios. Also, as demonstrated in Table 1, huge deviation in FE values for certain concentration scenarios indicates inability in perfectly controlling process parameters. Yet, repeatability of bad results for these concentration ratios implies that only the optimized parameters of the ratio 80:20% are of practical importance.

In the *J–E* plots, three different functions of an emitter can be observed: (1) current density saturation; (2) field emission; and (3) no emission. FE performance was enhanced in the case that graphene flakes were vertically oriented with respect to the substrate, when the emission was weakened, due to screening effects, over a critical rGO proportion.

Besides, FE performance can be further improved, in terms of low turn on field and high enhancement factor, by adopting three different approaches, according to the Fowler–Nordheim theory: (1) by shaping organized emission arrays, thus increasing the emitting area; (2) by selecting low work function *φ* materials, such as graphene-based materials; and (3) by increasing the geometrical aspect ratio of the emitter and therefore to improve the enhancement factor. On the one hand, as the polymer concentration in the composite emitter is increased, the relative concentration of graphene flakes in the polymeric matrix is decreased. Consequently, the density of the graphene flakes in the vacuum is also diminished. On the other hand, when the polymer content is very low, rGO flakes exhibit random protrusions, with reference to the substrate due to the Van der Waals forces, also resulting in limited emitting potential.

#### 3.1.3. Third Case: PCDTBT:PC_71_BM:rGO Composite Emitter

**Figure 7:**
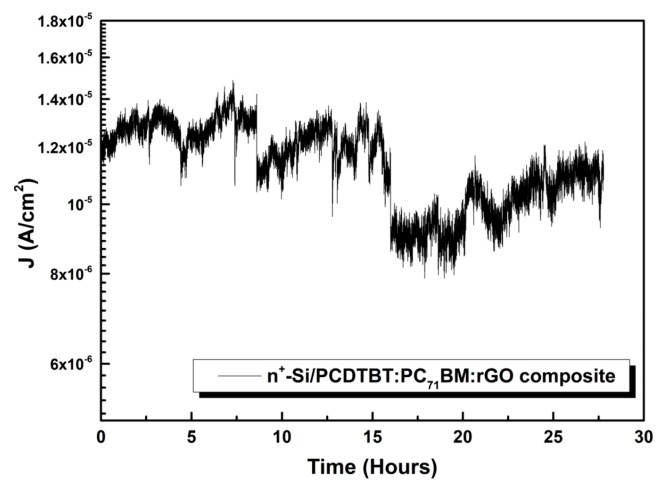
The evolution of the emission current density at a constant bias voltage of 1500 volts over a long period of continuous operation for the best rGO cathodes measured.

In a similar manner to the polymer:rGO case, a batch with 80% blend:20% rGO ink volume ratio gave the best results. Again, per each case, a set of five different ratio batches has been fabricated and tested, while each batch includes five fabricated cathode samples and only the best cathode results per batch are presented in Figure 5b. Accordingly, Figure 6 and Figure 7 depict a comparative chart of the best batches per each ratio with respect to the Fowler–Nordheim characterization and the overall FE performance parameters, respectively. All FE values per volume ratio set are included in Table 1.

#### 3.1.4. Field Emitter Stability

The evolution of the emission current density at a constant bias voltage of 1500 volts was tracked over a long period of over 25 h continuous operation for the best rGO-containing cathode that had been chosen among all samples. Figure 7 demonstrates the respectable stability of the emitter’s current density over time, resulting in a final drop of approximately 16% with regard to the initial current. These results explain why PCDTBT:PC_71_BM:rGO composite emitter has a higher level of long-term stability under the field emission experimental conditions related other rGO composite emitters that have been tested in the literature [34].

### 3.2. Morphological Characterization

**Figure 8:**
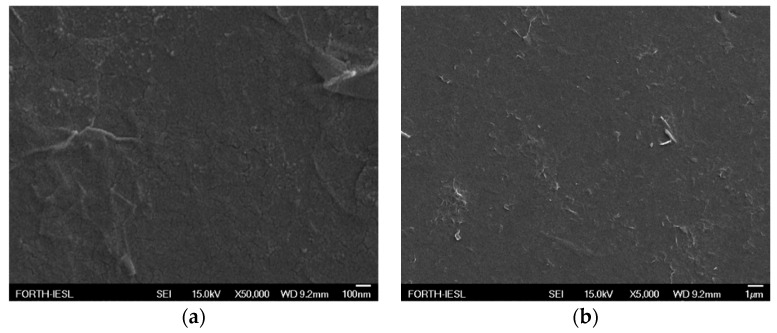
Top views of FE cathode surface with clearly visible edges in higher (**a**) and lower magnification scale (**b**). Distance among neighboring edges is remarkably close in the order of few microns or less.

**Figure 9:**
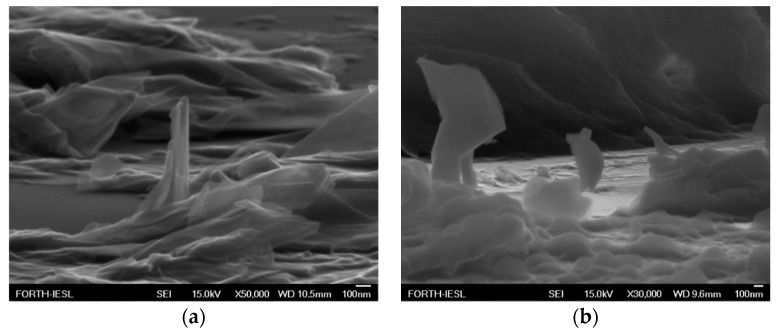
SEM images of a FE cathode edges in the cases of rGO bundles alone (**a**) and P3HT(80% *v*/*v*):rGO blend(20% *v*/*v*) (**b**). Thickness inhomogeneity due to the applied drop casting method has to be taken into account together with the formation of protruding edges. Impact of rGO sheets is clearly visible especially in (**a**).

Figure 8 and Figure 9 demonstrate scanning electron microscopy ((FESEM JEOL-JSM7000F) images which were extracted in order to verify the creation and morphology of extruding edges both prior and after FE.

## 4. Discussion

In general, HI/AcOH is by far more efficient by other reducing agents like hydrazine: optimum reduction dictates the compromise of yielding a partially reduced rGO. Although being of low occurrence, acid groups are present on rGO surface and result to a remaining polar nature of rGO, hence enabling rGO to form stable and homogeneous dispersions in THF [21].

As already mentioned in Section 2.4, the polymer–fullerene powder (PCDTBT:PC_71_BM) was unable to dissolve adequately in THF, therefore a mixture of DCB:CB was finally applied to dissolve successfully the solid components with the assistance of magnetic stirring. A possible explanation to this fact is that blend residues which are insoluble may probably contain the highest molecular weight fraction which could not be solubilized [57]. It can be said that high MW values adversely affect polymer solubility. Low solubility of polymers may direct to a strong interchain bonding or even chain aggregation. Seemingly, orthodichlorobenzene did not result to any further aggregation of PCDTBT chain, while it could simultaneously decelerate drying of the wet film [58]. Furthermore, the effect of THF additive in polymer and blend morphology mostly has to do with the solvent’s higher affinity for polymers than for PCBM. Diversifying the dipole moment by employing solvents similar to THF may control this affinity. Moreover, it has to be mentioned that THF, which has low boiling point, dissolves P3HT very easily, while such an easy dissolution for PCBM appears for the higher boiling point o-DCB, let alone the good solubility of PCDTBT [59].

With respect to the case of P3HT:rGO composite-based FE cathodes, among all different concentration variants, the volume ratio of 80% polymer:20% rGO ink gave the best results. FE trends are in accordance with relevant previous literature [21]. Indeed, component concentration significantly affects composite morphology and the dependent FE performance; Lower rGO flakes’ concentration inside the composite blend, reportedly, may favor the orientation of sheets at angles different to the planar substrate, hence inducing higher roughnesses [21]. As the P3HT:rGO ratio decreases, i.e., the polymer content increases, the rGO density into the polymer matrix decreases, giving rise to a corresponding decrease in the density of the rGO edges exposed to vacuum. On much higher rGO flakes’ concentration; however, screening of coated surface takes presumably place that deteriorates FE according to various studies [21,48,49]. Higher polymer content means fewer rGO sheets that are exposed to vacuum, while it has been demonstrated that thermal conductivity favors heat dissipation thus prolonging stability, in contrast with the case of bare rGO. On the other hand, lower polymer content allows rGO sheets to orientate themselves parallel to the substrate, hence resulting to fewer emitting edges and decreased FE performance. In general, at higher rGO ink concentrations, the base of the protruding bundles is a part of the rGO layer, whereas in the case of samples with high polymer content, bundles are sticking out of a polymer bulk. This observation suggests that there are two conduction processes present, conduction between rGO within flakes of a bundle and between polymer and sheets [50]. A final remark is that lower emitter edge density has been observed in high and low concentrations [21]. We conclude presuming that 80:20% analogy of P3HT:rGO better approaches the trade-off between screening and the density of protruding rGO edges, thus leading to better FE characteristics witnessed in Table 1, Figure 5 and Figure 6. 

The differences observed in field enhancement factor and in turn-on field between the composite emitters with different polymers (P3HT and PCDTBT) are directly related to their morphology instability and their different ionization state [60,61]. In this work, the highest field enhancement factor observed on semicrystalline p-type polymer P3HT and the lowest electrical threshold for amorphous p-type polymer of PCDTBT.

Besides, considering the third FE case of PCDTBT:PC_71_BM blends complementing rGO, it is already known that for blend containing OSCs a low temperature annealing at approximately 70 °C suffices for optimizing PCE, thus also blend morphology and phase separation [62,63]. The same logic is valid in the FE case as well, since charge carrier transfer is thereby optimized. It has been observed that the upper part of PCDTBT:PC_71_BM films is relatively enriched in PC_71_BM with negative gradient of fullerene concentration when examining deeper inside the annealed layer, yet in no case exceeds upper surface concentration of PC_71_BM the one of PCDTBT. Of course, PC_71_BM tends to reach the upper interface with air demonstrating a mild trend for phase separation, but PC_71_BM accumulation on the upper surface does not at all increase roughness due to PC_71_BM crystallites. Furthermore, a mild annealing at 70 °C neither impacts on PC_71_BM distribution towards depth, nor does it significantly affect blend crystallization, but enables the PCDTBT:PC_71_BM blend to self-organize into an optimal morphology for charge generation and extraction and, most importantly, eases the removal of trapped blend solvent [64]. Similar to the previous case of HI-reduced GO, the 20% rGO ink:80% polymer–fullerene blend ratio appears to yield optimized FE characteristics. The fact that rGO contributes best with the same concentration as in polymer:rGO, may indicate a nonsignificant impact of fullerene on final FE contrary to rGO. Yet, in accordance with not directly FE-relevant studies that include PC_71_BM [65] or PC_61_BM [56], the fullerene–rGO interaction is highlighted in terms of further improving charge transfer from the blend to emitting edges and a significant contact resistance reduction. Finally, it has to be noticed that in all cases research excluded rGO-free implementation of FE cathodes, since this research, tries to explain the electron emission from composites emitters with rGO flakes and polymer or charge transfer materials; such research would be out of the scope of current study.

## 5. Conclusions

In this study, four rGO-based field effect emitters were developed, optimized, and compared. rGO layers prepared by ΗΙ/AcOH reduction method and were coated on (a) n^+^ doped Si substrate, (b) n^+^-Si/P3HT:rGO, (c) n^+^-Si/PCDTBT:rGO, and (d) n^+^-Si/PCDTBT:PC_71_BM:rGO composites, respectively. We investigated the FE properties of different concentrations of polymer composite solutions to control the structural end electrical properties of the substrate. It is found that the cathodes based on PCDTBT:PC_71_BM:rGO displayed a field enhancement factor of ~2850, with remarkable stability over 25 h and low turn-on field in 0.6 V/μm.

The threshold field, enhancement factor and the remarkable stability of FE current were remarkably improved compared to the pristine Si demonstrating that is a promising FE cathode with potential applications in vacuum microelectronics and FEDs. 

Finally, taking into account the high potential of PCDTBT:PC_71_BM as successful blend candidate in conventional OSC devices, its optimized coating on a TCO or TCO/HTL substrate, either rigid or flexible, might result in significant number of photogenerated electrons and a further decrease in contact resistance that might further boost FE figures of merit in future, therefore possibly promising a new alternative type of photodetectors. 

## Figures and Tables

**Table 1:** Field enhancement factor and turn-on field figures classified per composite type (columns) and for HI/AcOH-reduced rGO ink with diversified ratios.

rGO Ratio (%)	n^+^-Si/P3HT:rGO		n^+^-Si/PCDTBT:rGO		n^+^-Si/PCDTBT:PC_71_BM:rGO	
	Field Enhancement β	Turn-on Field *E_to_* (V/μm)	Field Enhancement β	Turn-on Field *E_to_* (V/μm)	Field Enhancement β	Turn-on Field *E_to_* (V/μm)
100%	660 ± 10	1.60 ± 0.1	660 ± 10	1.60 ± 0.1	660 ± 10	1.60 ± 0.1
80%	300 ± 10	2.23 ± 0.1	170 ± 10	2.80 ± 0.1	80 ± 10	2.43 ± 0.1
60%	420 ± 10	2.05 ± 0.1	625 ± 10	2.40 ± 0.1	360 ± 10	2.16 ± 0.1
40%	915 ± 10	1.53 ± 0.1	1090 ± 10	1.58 ± 0.1	950 ± 10	1.53 ± 0.1
20%	2500 ± 10	1.03 ± 0.1	2050 ± 10	0.80 ± 0.1	2850 ± 10	0.60 ± 0.1

The ± values denote the standard deviation of each measured or estimated quantity.
